# A time-lapse embryo dataset for morphokinetic parameter prediction

**DOI:** 10.1016/j.dib.2022.108258

**Published:** 2022-05-11

**Authors:** Tristan Gomez, Magalie Feyeux, Justine Boulant, Nicolas Normand, Laurent David, Perrine Paul-Gilloteaux, Thomas Fréour, Harold Mouchère

**Affiliations:** a Nantes Université, Ecole Centrale Nantes, CNRS, LS2N, UMR 6004, F-44000 Nantes, France; b University of Nantes, Nantes University Hospital, Inserm, CNRS, SFR Santé, Inserm UMS 016, CNRS UMS 3556, F-44000 Nantes, France. Adress: 8 Quai Moncousu, 44007 Nantes; c University of Nantes, Nantes University Hospital, Inserm, DSN, Inserm UMR 1064, F-44000 Nantes, France. Adress: 8 Quai Moncousu, 44007 Nantes; d University of Nantes, Nantes University Hospital, Inserm, CRTI, Inserm UMR 1064, F-44000 Nantes, France. Adress: 8 Quai Moncousu, 44007 Nantes

**Keywords:** Human reproduction, Deep learning, Time-lapse, Videos, IVF, Computer vision

## Abstract

One of the most common treatments for infertile couples is In Vitro Fertilization (IVF). It consists of controlled ovarian hyperstimulation, followed by ovum pickup, fertilization, and embryo culture for 2–6 days under controlled environmental conditions, leading to intrauterine transfer or freezing of embryos identified as having a good implantation potential by embryologists. To allow continuous monitoring of embryo development, Time-lapse imaging incubators (TLI) were first released in the IVF market around 2010. This time-lapse technology provides a dynamic overview of embryonic in vitro development by taking photographs of each embryo at regular intervals throughout its development. TLI appears to be the most promising solution to improve embryo quality assessment methods, and subsequently the clinical efficiency of IVF. In particular, the unprecedented high volume of high-quality images produced by TLI systems has already been leveraged using modern Artificial Intelligence (AI) methods, like deep learning (DL).

An important limitation to the development of AI-based solutions for IVF is the absence of a public reference dataset to train and evaluate deep learning (DL) models. In this work, we describe a fully annotated dataset of 704 TLI videos of developing embryos with all 7 focal planes available, for a total of 2,4M images. Of note, we propose highly detailed annotations with 16 different development phases, including early cell division phases, but also late cell divisions, phases after morulation, and very early phases, which have never been used before. This is the first public dataset that will allow the community to evaluate morphokinetic models and the first step towards deep learning-powered IVF. We postulate that this dataset will help improve the overall performance of DL approaches on time-lapse videos of embryo development, ultimately benefiting infertile patients with improved clinical success rates.

## Specifications Table

**Table:** 

Subject	Computer Vision and Pattern Recognition
Specific subject area	Time-lapse videos of human embryos with morphokinetic parameter annotations.
Type of data	ImageVideo
How the data were acquired	Images were acquired using the Embryoscope©, a time-lapse incubator system (Vitrolife©, Sweden) with a camera under a 635 nm LED light source passing through Hoffman's contrast modulation optics.
Data format	RawAnnotated
Description of data collection	The data comes from infertile couples that underwent Intracytoplasmic Sperm Injection (ICSI) cycles. Patient treatment and embryo culture protocol were described in a previous study [1]. Videos corresponding to embryos with less than 6 annotated phases were rejected to keep only videos with detailed annotations. Images were extracted using the manufacturer's (Vitrolife©) API.
Data source location	• Institution: Nantes University Hospital • City/Town/Region: Nantes • Country: France
Data accessibility	Repository name:The dataset is hosted at https://zenodo.org[2]Data identification number: 10.5281/zenodo.6390798Direct URL to data: https://doi.org/10.5281/zenodo.6390798
Related research article	No related article

## Value of the Data

• This dataset is composed of 704 TLI videos of developing embryos with all 7 focal planes available, for a total of 2,4M images.
• We propose highly detailed annotations with 16 different development phases, including early cell division phases, but also late cell divisions, phases after morulation, and very early phases, which have never been used before.
• This dataset can be used to train machine learning models to identify the various phases of embryo development from polar body appearance to blastocyst hatching.
• Researchers and developers of time-lapse automated analysis software can use this dataset to evaluate and compare new models against previously proposed models.
• To the best of our knowledge, this is the first embryo time-lapse dataset publicly available.

## 1 Data Description

This dataset is composed of 704 videos, each recorded at 7 focal planes, accompanied by the annotations of 16 cellular events.

First, we describe how the events were annotated, then how each frame was assigned a label, followed by the file descriptions and some dataset statistics and samples.

**Dataset annotation.** Each video was annotated by a qualified and experienced embryologist undergoing regular internal quality control. For each video, the annotation consists of the timing of 16 cellular events noted tPB2, tPNa, tPNf, t2, t3, t4, t5, t6, t7, t8, t9+, tM, tSB, tB, tEB, and finally tHB. We use the definition of the events proposed by Ciray et al. [3]: polar body appearance (tPB2), pronuclei appearance and disappearance (tPNa and tPNf), blastomere division from 2-cell stage to 9 (and more) cells-stage (t2,t3,t4,t5,t6,t8 and t9+), compaction (tM), blastocyst formation (tSB, tB), expansion and hatching (tEB and tHB). We chose to use more events than previous work [4], [5], [6], [7] to develop models that can more precisely describe embryo development in a controlled environment.

We started prospective annotation of the database according to this reference work in 2014, while annotations made before 2014 were retrospectively checked.

**From event timing to frame labels.** We formulate the task as an image classification problem. This means that we need to assign a label to each frame that the model will be trained to predict. However, the annotations given by the biologists are timings in hours post-fertilization that indicates the temporal position of events in the video.

**Fig. 1:**
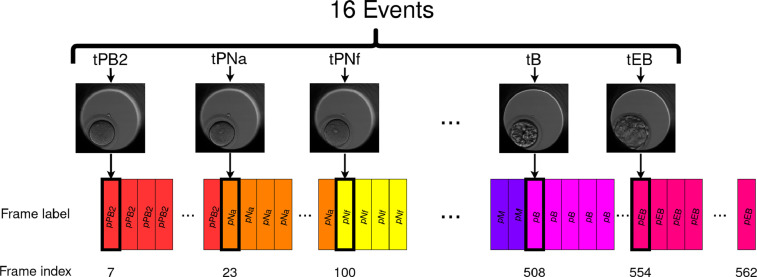
The method used to assign a label to every frame of the video. First, we identify at which frame each event occurs and assign to these frames a label corresponding to the event they show. The other frames are assigned the label corresponding to the most recent event that has occurred in the previous frames. Note that all frames are labeled except the frames before tPB2 as they precede all the events. The video used as an example here is AG274-2.

Knowing the timing at which each frame was taken, we identify the frames corresponding to each event and assigns them a label corresponding to the event they show (noted pPB2, pPNa, pPNf, p2, p3, p4, p5, p6, p7, p8, p9+, pM, pSB, pB, pEB or pHB), as illustrated in Fig. 1.

The other frames are assigned the label corresponding to the most recent event that has occurred in the previous frames. This labeling constructs the succession of embryo development phases, delimited by the cellular events.

**Dataset files.** The dataset is composed of several compressed folders:

- *embryo_dataset.tar.gz* contains 704 folders, each containing all the frames of one video. An embryo is a 3D object and the focal plane of the microscope can be changed to better visualize the embryo. This compressed folder contains images recorded at the central focal plane called F0. The images are JPEG files in shades of gray with a 500 × 500 resolution.
- *embryo_dataset_annotations.tar.gz* contains the 704 CSV annotations files. Each CSV is the annotation of one video and has three columns: phase, start frame index and end frame index that indicates the frame interval of each phase. Table 1 shows an example with the annotation file of the video AG274-2, which construction is illustrated in Fig. 1.

**Table 1:** Example of an annotation CSV file. The first column indicates the phase (the label to predict) and the second and third columns indicate the frame index at which this phase starts and ends. Note that all frames are labeled except the frames before tPB2 as they precede all the events. The file shown here is the annotation of the video AG274-2 which is called AG274-2_phases.csv.

tPB2	7	22
tPNa	2	99
tPNf	100	111
t2	112	169
t3	170	171
t4	172	177
t5	178	228
t6	229	234
t7	235	318
t8	319	328
t9+	329	464
tM	465	491
tSB	492	507
tB	508	553
tEB	554	562
- Six other compressed folders containing the same videos as embryo_dataset.tar.gz, except recorded with different focal plane settings. There is 6 alternative focal planes setting available: F-45, F-30, F-15, F15, F30 and F45. The folders are named as follows *embryo_dataset_X.tar.gz* where X is one of the mentioned focal planes. Each folder contains 704 folders, each containing all the frames of one video, recorded at the focal plane X.

**Fig. 2:**
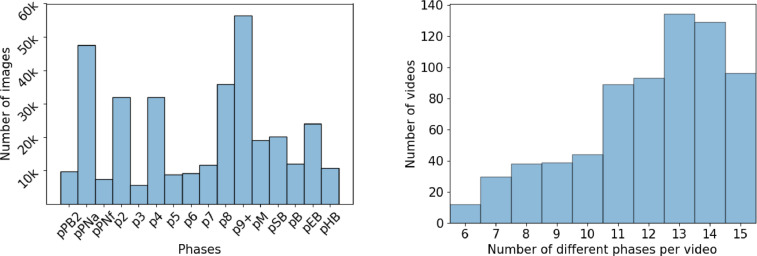
Statistics of the dataset. (a) The number of images per phase in the dataset. (b) Distribution of the number of phases per video in the dataset.

**Dataset statistics.** Deep learning models are heavily dependent on data and might provide poor performance on a specific class if the amount of input corresponding to it is too small. This is why for each label, we provide at least several thousand images (Fig. 2 (a)). Most videos have at least 8 annotated phases and approximately 360 videos have more than 13 phases annotated, illustrating the richness of annotation of our dataset (Fig. 2 (b)).

**Fig. 3:**
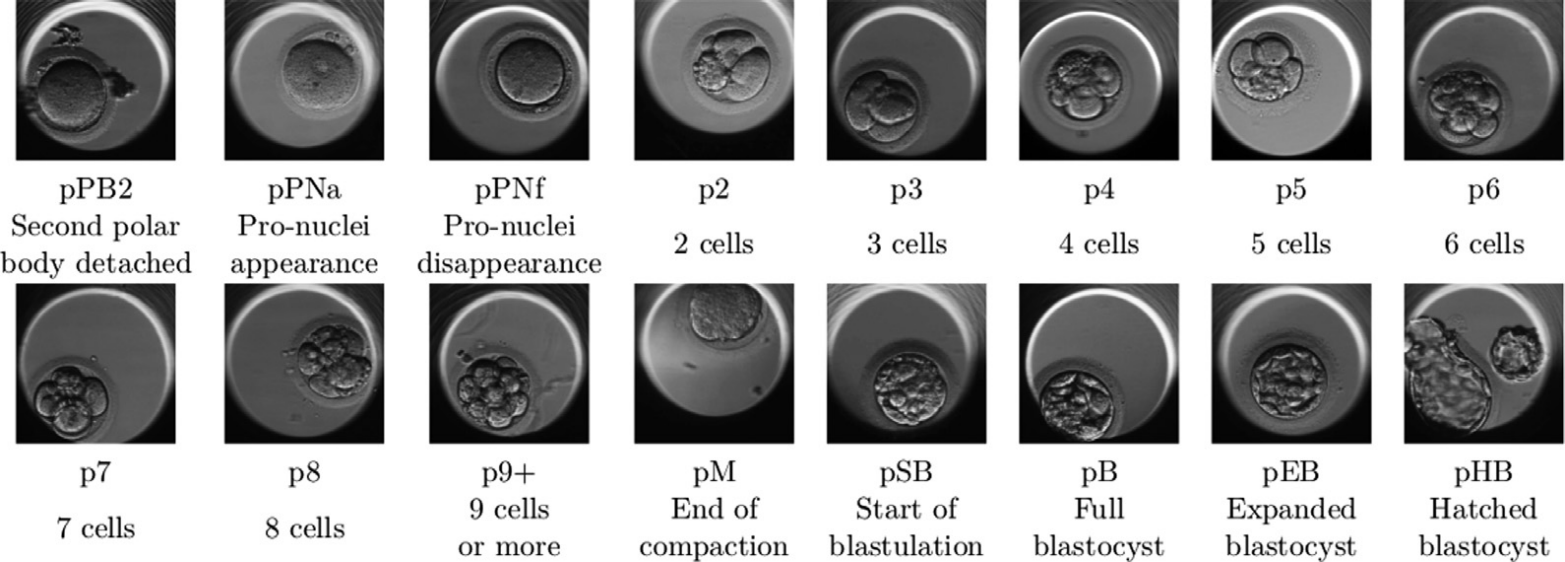
Illustrations of the 16 development phases used.

**Dataset samples.** Sample images allow one to have a clear view of the content of the dataset and the annotations associated with the images (Fig. 3). Note that, depending on their position in the well, embryos can sometimes be partially occluded which is quite common in time-lapse videos. However, even when a part of the embryo is hidden, the images are sufficient to identify the development phase.

## 2 Experimental Design, Materials and Methods

Between 2011 and 2019, 716 infertile couples underwent Intracytoplasmic Sperm Injection (ICSI) cycles in our University-based IVF center and had all their embryos cultured and monitored up to blastocyst stage with a TLI system. We acknowledge that only ICSI cycles were included in our time-lapse devices over that period, as we considered that conventional IVF would lead to different developmental timings as compared to ICSI. We do not routinely use assisted hatching. There were no major lab changes over the study period. Patient treatment and embryo culture protocol were described in a previous study [1]. In brief, embryo culture was performed from fertilization (day 1) up to blastocyst stage (day 5 or day 6) at 37 °C with 5% O2 and 6% CO2 in a sequential culture medium, i.e. G1 plus (Vitrolife©, Sweden) from day 0 to day 3, followed by G2 plus (Vitrolife©, Sweden). We acknowledge that culture media might impact embryo development and have an evolving composition throughout embryo development. However, the available literature does not support the concept of medium-dependent morphokinetic patterns [8]. Although we agree that there is a need to clarify IVF culture media composition to enhance our understanding of embryo development [9], there is no evidence to our knowledge that the content of commercial culture media changes over time in ways that are important enough to consider. The images were acquired with a TLI system (Embryoscope©, Vitrolife©, Sweden) every 10 to 20 min by a camera under a 635 nm LED light source passing through Hoffman's contrast modulation optics.

To select the videos, we first excluded videos with strictly less than 6 phases annotated to keep only videos with highly detailed annotations and then randomly selected 10% of the remaining videos, which constitutes a dataset of 704 videos. Among these videos, 499 correspond to embryos considered to be morphologically viable and subsequently chosen for transfer, while the other videos correspond to discarded embryos because of poor development. These discarded embryos allowed us to study a variety of abnormal embryonic features (abnormal morphology, abnormal fertilization/number of pro-nuclei, necrosis, fragmentation, developmental delay, etc.) or problems during image acquisition (sharpness, change of focus, brightness, etc.). We subsequently extracted all images using the Application Programming Interface (API) provided by the TLI manufacturer (Vitrolife©). The information about embryo viability is not included in this dataset as the purpose is to focus solely on morphokinetic parameter prediction.

## Ethics Statements

The Local Institutional Review Board (GNEDS) (local ethics committee) approved this project before it started. The anonymised database is registered under CNIL approval number

1760497. All patients gave informed consents for the use of their anonymized clinical data in observational study. The research has been carried out in accordance with The Code of Ethics of the World Medical Association (Declaration of Helsinki).

## CRediT Author Statement

**Tristan Gomez:** Methodology, Software, Data curation, Writing, Original Draft Supervision; **Magalie Feyeux:** Data curation, Writing; **Justine Boulant:** Software; **Nicolas Normand:** Reviewing; **Laurent David:** Supervision, Reviewing; **Perrine Paul-Gilloteaux:** Supervision, Reviewing; **Thomas Fréour:** Supervision, Reviewing; **Harold Mouchère:** Supervision, Reviewing.

## Declaration of Competing Interest

The authors declare that they have no known competing financial interests or personal relationships that could have appeared to influence the work reported in this paper.

## Data Availability

Human embryo time-lapse video dataset (Original data) (Zenodo).
